# A Communication from the Dental Protective Association of the United States

**Published:** 1891-06

**Authors:** J. N. Crouse


### CORRESPONDENCE.

### A Communication from the Dental Protective Association of the United States.

DEAR SIR:—

The enclosed is a brief review and summary of the recent litigation of the International Tooth Crown Company vs. Edward S. Gaylord et al, recently ended by a unanimous decision from all the Judges of the Supreme Court of the United States, in which the Richmond Crown patent and Richmond patent on Preparation of Roots for Crowns, were declared invalid. This is the case that the Dental Protective Association of the United States took up and defended in the Supreme Court, in association with, and at the request of Dr. Northrop's committee. I will state that by the result of this decision all forms of Richmond Crowns are now open to the dental profession for manufacture and free use, as well as the Richmond method or process for preparing roots for his crowns, which was a patent for freezing and cutting off the tooth and driving the pulp out and immediately filling the end of the root at one operation. The decision of the court occupies a number of printed pages, but in substance is as follows:

FIRST. That what Richmond had done before the taking out of his crown patents disclosed substantially whatever invention there was in his crown, or process of making, and this invention had been in successful use over two years before being patented, consequently had been dedicated to the public. Some later changes in construction were made the claim for sustaining the patent; the claim being that only after these changes were made was the invention a success, that it was in use only as an experiment before, to which the Court said:

SECOND. That whatever changes were made as appearing in these patents, from what existed before and was known to modern dentists, was not invention in the eye of the patent law but simply the mechanical skill of a skillful dentist.

While this decision is of incalculable value to the dental profession it must not be forgotten that the decision does not end, by any manner or means, the litigation between the International Tooth Crown Company and the dental profession. That company owns some twenty-five or thirty other patents relating to dentistry, some of these being in suit. One of these patents known to the profession as the "Low Bridge" was sustained in Judge Wallace's United States Court in the Southern District in New York some four or five years ago, and before the Dental Protective Association of the United States was organized. And the Dental Protective Association is now engaged in an extended and expensive contest in the Federal Courts to secure its defeat and to have the same judicial declaration of invalidity declared against it by the United States Courts as has just been obtained upon the cases referred to.

We stated in our former circular that any one not a member of the association before May 15th, if sued after that date on the Richmond Crown Patents would not receive the defence of the association, but as we have wiped out the Richmond Crown patents, this limit to membership is removed and any one not a member can still have an opportunity of joining. We feel justified in saying that it is asking but a little of each one to send in the ten dollars membership fee, and to do their part in this great work, which is so well begun, of freeing us from bondage. The next great battle which we now have with the enemy is defeating them in suits brought upon the Low Bridge Patent which will be made the basis of another circular and communication with full information, within a short time.

Hoping for a favorable response from each one, I remain,

Yours truly, J. N. Crouse, Chairman.

---

"Disuse is a predisposing cause of decay in the teeth. In my practice I have always advised my patients, where they had teeth decaying rapidly, to eat their food as dry as possible, to masticate it thoroughly, and to give the teeth as much use as they could, and that little advice to my patients has been of great assistance to me and of very great advantage to the patients themselves." Prof. C. N. Pierce.

